# 7-ketocholesterol and 27-hydroxycholesterol decreased doxorubicin sensitivity in breast cancer cells: estrogenic activity and mTOR pathway

**DOI:** 10.18632/oncotarget.19789

**Published:** 2017-08-02

**Authors:** Chun-Wei Wang, Chiung-Chiao Huang, Pei-Hsin Chou, Yu-Ping Chang, Shouzuo Wei, Frederick Peter Guengerich, Yueh-Ching Chou, Sheng-Fan Wang, Ping-Shan Lai, Pavel Souček, Yune-Fang Ueng

**Affiliations:** ^1^ National Research Institute of Chinese Medicine, Ministry of Health and Welfare, Taipei, Taiwan, R.O.C; ^2^ Institute of Biopharmaceutical Sciences, School of Pharmacy, National Yang-Ming University, Taipei, Taiwan, R.O.C; ^3^ Department of Pharmacology, School of Medicine, National Yang-Ming University, Taipei, Taiwan, R.O.C; ^4^ Department of Environmental Engineering, National Chung-Kung University, Tainan, Taiwan, R.O.C; ^5^ Department of Medicine, Vanderbilt University School of Medicine, Nashville, TN, USA; ^6^ Department of Biochemistry, Vanderbilt University School of Medicine, Nashville, TN, USA; ^7^ Department of Pharmacy, Taipei Veterans General Hospital, Taipei, Taiwan, R.O.C; ^8^ Department of Chemistry, College of Science, National Chung-Hsin University, Taichung, Taiwan, R.O.C; ^9^ Department of Pharmacy, Taipei Medical University, Taipei, Taiwan, R.O.C; ^10^ Institute of Medical Sciences, Taipei Medical University, Taipei, Taiwan, R.O.C; ^11^ Department of Toxicogenomics, National Institute of Public Health, Prague, Czech Republic

**Keywords:** 7-ketocholesterol, 27-hydroxycholesterol, doxorubicin, P-glycoprotein, estrogen receptor

## Abstract

Hypercholesterolemia is one of the risk factors for poor outcome in breast cancer therapy. To elucidate the influence of the main circulating oxysterols, cholesterol oxidation products, on the cell-killing effect of doxorubicin, cells were exposed to oxysterols at a subtoxic concentration. When cells were exposed to oxysterols in fetal bovine serum-supplemented medium, 7-ketocholesterol (7-KC), but not 27-hydroxycholesterol (27-HC), decreased the cytotoxicity of doxorubicin in MCF-7 (high estrogen receptor (ER)α/ERβ ratio) cells and the decreased cytotoxicity was restored by the P-glycoprotein inhibitor verapamil. 7-KC stimulated the efflux function of P-glycoprotein and reduced intracellular doxorubicin accumulation in MCF-7 but not in ERα(-) MDA-MB-231 and the resistant MCF-7/ADR cells. In MCF-7 cells, 7-KC increased the mRNA and protein levels of P-glycoprotein. The 7-KC-suppressed doxorubicin accumulation was restored by the fluvestrant and ERα knockdown. In a yeast reporter assay, the ERα activation by 7-KC was more potent than 27-HC. 7-KC, but not 27-HC, stimulated the expression of an ER target, Trefoil factor 1 in MCF-7 cells. When charcoal-stripped fetal bovine serum was used, both 7-KC and 27-HC induced Trefoil factor 1 expression and reduced doxorubicin accumulation in MCF-7 cells. 7-KC-reduced doxorubicin accumulation could be reversed by inhibitors of phosphatidylinositol 3-kinase, Akt, and mammalian target of rapamycin (mTOR). These findings demonstrate that 7-KC decreases the cytotoxicity of doxorubicin through the up-regulation of P-glycoprotein in an ERα- and mTOR-dependent pathway. The 7-KC- and 27-HC-elicited estrogenic effects are crucial in the P-glycoprotein induction in breast cancer cells.

## INTRODUCTION

Breast cancer is the most common cancer in women worldwide and the 4th most common cancer-related death in Taiwan (“Cancer registry annual report, 2012” reported by Health Promotion Administration, Ministry of Health and Welfare (Taiwan) in 2015). The breast is primarily a mix of fat and glands, with the volume and weight ratios of fat to total breast varying from 7% to 56% and 4% to 38%, respectively [1]. Breast cancer is prevalent in postmenopausal women who are obese or undergoing hormone treatment [2]. Patients with a greater body mass index (BMI) had less-favorable outcomes than lean women, particularly in hormone-receptor-positive patients [3]. Breast cancer patients with higher blood cholesterol had poorer therapeutic outcome [4]. More evidence showed that intrinsic factors, e.g. hypoxia and elevated systemic oxidative stress, exist in the tumor microenvironment, creating resistance to chemotherapy [5, 6]. Oxysterols are known to be generated from cholesterol oxidation either by autooxidation or cytochrome P450 isoforms [7]. 4-Hydroxycholesterol (HC), 7-HC, 7-ketocholesterol (7-KC), 25-HC and 27-HC are the main oxysterols identified in human samples (Table 1). The contents of each oxysterol in human plasma and tissue samples showed variations in the reports. The B-ring oxysterols, 7-HC and 7-KC, were identified as the markers of oxidative stress *in vivo* [5, 8]. The elevated blood levels of 7-KC and 27-HC were observed in patients with cancer and inflammation [5, 7, 9, 10]. 27-HC was the most abundant oxysterol in most human blood samples and promoted tumor development in ovariectomized breast cancer mouse models [7, 11]. 7-KC can be generated from the oxidation of cholesterol, 7-HC and 7-dehydrocholesterol [12]. Blood 7-HC level was comparable to 27-HC [7] (Table 1). Serum 7-KC concentrations in lung and rectal cancer patients were 2- to 3-fold higher than those in healthy participants [10]. Although there was no significant difference in serum cholesterol and 27-HC levels between the benign control and breast cancer patients, the mean 27-HC level in normal breast tissues in breast cancer patients was 3-fold higher than in the control group [13]. In breast cancer patients, the 27-HC level was 2-fold higher in tumor than in normal breast tissues. The increased tumor levels of oxysterols, such as 7-KC and 27-HC can be risk factors for the poor outcome in cancer therapy.

**Table 1:** Concentrations/contents of oxysterols in human plasma and tissues

Sterol	Disease status	Blood, μM	Tissue	Tissue content
4β-HC	Healthy^[1-5]^	0.07 ± 0.02 - 0.191 ± 0.10		
	Non-alcoholic fatty liver disease^[3]^	0.20 ± 0.02		
7α-HC	Healthy^[1, 2, 4-8]^	0.03 - 0.62		
	Obese^[6]^	0.48 ± 0.00		
	Hypercholesterolemia^[7]^	0.10 ± 0.01		
	Diabetes^[7]^	0.08 ± 0.01		
7-KC	Healthy^[4-10]^	0.06 ± 0.00 - 5.0		
	Obese^[6]^	2.44 ± 0.01		
	Hypercholesterolemia^[7, 8, 11]^	0.06 ± 0.04 - 0.13 ± 0.02, 7.0		
	Diabetes^[7]^	0.08 ± 0.10		
	Cancers^[12], a^	0.18 - 0.82		
	Chronic hepatitis/ cirrhosis^[13]^	0.023 ± 0.013 - 0.052 ± 0.025	normal liver	0.24 ± 0.11μg/g liver (2.38 ± 1.41 ng/mg protein)
			cirrhosis liver	0.29 ± 0.47μg/g liver (4.03 ± 6.81 ng/mg protein)
25-HC	Healthy^[1, 2, 5, 7]^	<0.01 - 0.09		
	Hypercholesterolemia^[7]^	0.00050 ± 0.00002		
	Non-alcoholic fatty liver disease^[3]^	0.14 ± 0.01		
	Diabetes^[7]^	0.00047 ± 0.00005		
27-HC	Healthy^[1-3, 7, 8, 14, 15]^	0.012 ± 0.001 - 0.38 ± 0.11, 0.22 - 0.60	benign normal breast	0-1 ng/mg protein
	Hypercholesterolemia^[7]^	0.021 ± 0.001		
	Non-alcoholic fatty liver disease^[3]^	0.62 ± 0.03		
	Diabetes^[7]^	0.025 ± 0.002		
	Breast cancer^[15]^	∼ 0.335^b^	normal breast	0-8 ng/mg protein
			breast tumor	0-18 ng/mg protein

^a^Serum samples were from one rectum cancer, four lung cancer and one mesothelioma patients. References: ^**[1]**^J. Lipid Res., 50:350-357, 2009; ^**[2]**^J. Lipid Res., 36:2275-2281, 1995; ^**[3]**^J. Gastroenterol., 47:1257-1266, 2012; ^**[4]**^Physiol. Rev., 80:361-466; ^**[5]**^J. Steroid Biochem. Mol. Biol., https://doi.org/10.1016/j.jsbmb.2015.11.017, 2015; ^**[6]**^J. Clin. Endocrinol. Metab., 93:4282-4289, 2008; ^**[7]**^Lipids, 35, 333-338, 2000; ^**[8]**^Anal. Biochem., 225:73-80, 1995; ^**[9]**^Free Radic. Biol. Med., 42:698-705, 2007; ^**[10]**^ Free Radic. Biol. Med., 39:1152-1161, 2005; ^**[11]**^Free Radic. Biol. Med., 17:397-409, 1994; ^**[12]**^Tohoku J. Exp. Med., 140:245-258, 1983; ^**[13]**^Liver Transpl., 11:1494-1504, 2005; ^**[14]**^J. Lipid Res., 45:776-781, 2004; ^**[15]**^Cell Rep., 5:637-645, 2013.

Doxorubicin (adriamycin, ADR) is currently used in the first-line neoadjuvant and adjuvant chemotherapy regimens for breast cancer patients [14]. The efflux pumps belong to the ATP-binding cassette (ABC) superfamily and are responsible for the extrusion of a variety of xenobiotics and endogenous substances. Doxorubicin has been reported to be a substrate of efflux transporters including P-glycoprotein (P-gp) (MDR1 or ABCB1-encoded), multidrug resistance-associated proteins (MRP) (ABCC-encoded) and a breast cancer resistant protein (BCRP) (ABCG2-encoded) [15, 16]. One of the main causes of the therapeutic failure of doxorubicin is drug resistance resulting from the upregulation of the efflux transporters. The induction of transporters confers intrinsic resistance to chemotherapeutic agents before the drugs are taken and acquired resistance after repeated treatment with chemotherapeutic agents [17, 18].

In breast cancer patients, use of the cholesterol-lowering agent simvastatin did not affect blood estradiol (E2) and estrone concentrations, whereas simvastatin treatment reduced the blood concentration of estone sulfate (a substrate of MRP and BCRP, [16, 19]) in female breast cancer patients, especially in postmenopausal participants [20]. Another lipid-lowering agent, atorvastatin, downregulated the expression of MDR1 in HepG2 and human peripheral blood mononuclear cells [21]. The use of atorvastatin significantly decreased the plasma concentration of 7-KC [7]. Our previous study of Huh-7 hepatoma cells showed that exposure to 7-KC increased the efflux function and the protein level of P-gp through the phosphatidylinositol 3-kinase (PI3K)/mammalian target of rapamycin (mTOR) phosphorylation signaling pathway [22]. Additionally, 20- and 25-HC (2.5-10 μM) were reported to induce the murine cholesterol export pump ABCG1 mRNA level in RAW264.7 cells [23]. To examine the involvement of oxysterols in drug resistance, effects of oxysterols on the cell-killing effect of doxorubicin were studied in MCF-7 (human breast adenocarcinoma, high estrogen receptor (ER)α(+)/ERβ(+) ratio) cells. The oxysterols and transporters responsible for the reduced cytotoxicity of doxorubicin were identified. The alterations in MCF-7 cells were compared to T47D (human breast ductal carcinoma, estrogen receptor low ERα(+)/ERβ(+) ratio) [24], MB-231 cells (human breast adenocarcinoma, ERα(-)) and doxorubicin-resistant MCF-7/ADR cell lines. The involvement of ERα and signaling pathways in the 7-KC-mediated decrease of doxorubicin accumulation was examined.

## RESULTS

### Cytotoxicities of oxysterols in breast cancer cell lines

To reduce the interference of serum factors on the cell-killing effect of doxorubicin and to minimize the stress-response induced by complete removal of the serum factors [25], cell growth of MCF-7 cells in the presence of different concentrations of fetal bovine serum (FBS) was monitored using the MTT and trypan blue exclusion assays (data not shown). When the FBS concentration was reduced from 10% to 2%, there was no obvious change of cell growth. As monitored by trypan blue staining, cell survival was significantly decreased by 10% in MCF-7 cells cultured in a medium without serum supplement. Thus, cells were exposed to oxysterols (stock solution in ethanol) in the medium supplemented with 2% FBS (v/v, ∼10 pM E2 in medium).

The average blood and tissue concentrations of oxysterols reported in patients or healthy participants (Table 1) were generally below their cytotoxic concentrations. To prevent the interference of cytotoxicities of oxysterols on the study of the cell-killing effect of doxorubicin, cytotoxicities of oxysterols were determined in MCF-7 cells. As monitored by the MTT assay, 48-h exposure of MCF-7 cells to 4β- and 7α-HC decreased cell growth at a concentration higher than 15 μM and 10 μM, respectively (Supplementary Figure 1A). 25-HC showed cytotoxicity at a concentration of ≥ 7.5 μM. 27-HC caused a biphasic change in cell growth. Cell growth was stimulated by 27-HC at 0.1 μM but decreased at ≥ 5 μM. Like 27-HC, 7-KC caused a biphasic change of cell growth (Supplementary Figure 1B). Cell growth was stimulated by 7-KC at 2.5 μM but decreased at ≥ 10 μM. For the purpose of comparison, cytotoxicity of 7-KC was further examined in T-47D, MB-231, and MCF-7/ADR cells. Cell growth was stimulated by 5 μM 7-KC in T-47D cells. 7-KC decreased cell growth at ≥ 15 and ≥ 30 μM in T-47D and MB-231 cells, respectively. Growth of MCF-7/ADR cells was stimulated by 7-KC at 2.5 μM but decreased when the exposure concentration was greater than 10 μM. 7-KC caused a biphasic change of cell growth not only in MCF-7, but also in T-47D and MCF-7/ADR cells. In the following studies, breast cancer cell lines were exposed to oxysterols at concentrations below respective growth-inhibition concentrations (subtoxic concentration). The subtoxic concentrations of 4β-HC, 7α-HC, 7-KC, 25-HC and 27-HC were below 15, 10, 10, 2 and 5 μM, respectively.

### Effects of oxysterols on the cell-killing effect of doxorubicin in breast cancer cell lines

**Figure 1:**
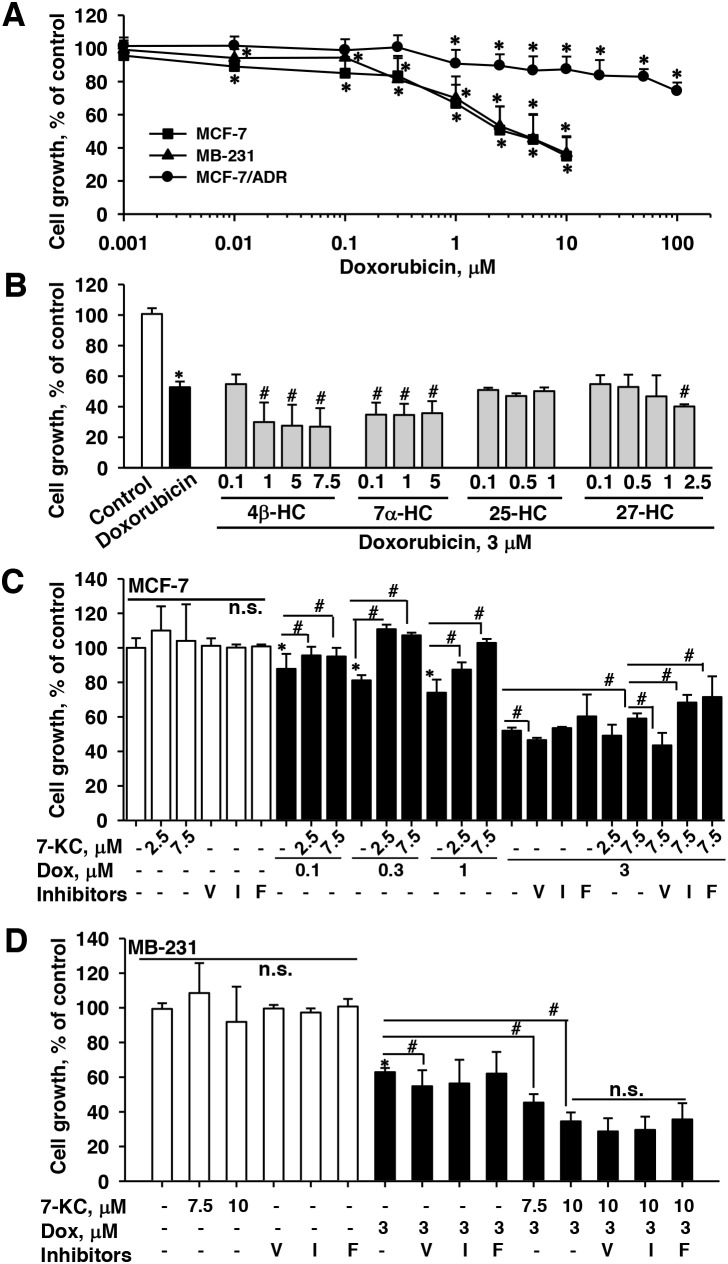
Effects of oxysterols and transporter inhibitors on the cell-killing effect of doxorubicin in breast cancer cells Cell growth was monitored by measuring mitochondrial MTT reduction activity. Panel **(A)** shows the cell-killing effects of increasing concentrations of doxorubicin in MCF-7, MB-231, and MCF-7/ADR cells. Panel **(B)** shows the alterations of the cell-killing effect of doxorubicin by hydroxycholesterols (HCs) in MCF-7 cells. Panels **(C)** and **(D)** show the alterations of the cell-killing effect of doxorubicin by 7-ketocholesterol (7-KC) and transporter inhibitors in MCF-7 and MB-231 cells, respectively. Cells were exposed to oxysterols and transporter inhibitors for 24 h prior to the co-exposure to 3 μM doxorubicin and respective oxysterols for a further 24 h. The exposure concentrations of transporter inhibitors were 10 μM verapamil (V), 5 μM indomethacin (I), and 1 μM fumitremorgin C (F). The results are presented as means ± SD of three independent experiments with three determinations within each experiment. n.s.: There was no significant difference compared to the control vehicle exposure or cells without the exposure to inhibitors. *p < 0.05, compared to the vehicle control. ^**#**^p < 0.05, compared to the doxorubicin-treated cells or the comparison between two treatments as indicated.

In MCF-7 cells, 24-h exposure to doxorubicin decreased cell survival with an IC_50_ value of 3.3 ± 0.5 μM (Figure 1A). In MB-231 cells, the IC_50_ value for the cell-killing effect of doxorubicin was 3.7 ± 0.3 μM, close to that for killing MCF-7 cells. In MCF-7/ADR cells, cells showed resistance to doxorubicin cytotoxicity. Although the growth of MCF-7/ADR cells was significantly reduced by doxorubicin at a concentration >1 μM, mild reduction (24%) of cell growth was observed when the doxorubicin concentration was increased to 100 μM (IC_50_ > 100 μM). According to the IC_50_ values, MCF-7 and MB-231 cells were exposed to 3 μM doxorubicin to examine the changes of the cell-killing effect of doxorubicin by oxysterols. MCF-7/ADR cells were exposed to a relatively higher doxorubicin concentration of 10 μM. Cells were pre-exposed to an oxysterol for 24 h and then co-exposure to an oxysterol and doxorubicin for further 24 h. In MCF-7 cells, pre-exposure to 4β-, 7α- and 27-HC enhanced the cell-killing effect of doxorubicin, whereas 25-HC did not affect the cytotoxicity induced by exposure to doxorubicin (Figure 1B). Among oxysterols, only 7-KC significantly reduced the cell-killing effect of doxorubicin at 3 μM (Figure 1C). In female patients with breast cancer, with intravenous infusion of doxorubicin at 60 mg/m^2^ body surface area, the mean plasma concentrations of doxorubicin are 1.1 and 0.6 μM in normal and overweight patients, respectively [26]. When reducing the exposure concentration of doxorubicin to 0.1, 0.3 and 1 μM, the cytotoxicity of doxorubicin was also significantly suppressed by the co-exposure to 2.5 and 7.5 μM 7-KC in MCF-7 cells.

In MCF-7 cells, the 7-KC-reduced doxorubicin cytotoxicity could be recovered by the co-exposure to a P-gp inhibitor verapamil, whereas this reduction was neither affected by indomethacin (an inhibitor of MRP2) nor by fumitremorgin C (an inhibitor of BCRP). Like the response in MCF-7 cells, the co-exposure to verapamil increased the cytotoxicity of doxorubicin in MB-231 cells. However, 7.5 and 10 μM 7-KC enhanced the cell-killing effect of doxorubicin and none of the inhibitors of P-gp, MRP2 and BCRP caused significant changes to the enhanced cell-killing effects by 7-KC in MB-231 cells (Figure 1D). In MCF-7/ADR cells, the cell-killing effect and accumulation of doxorubicin were not affected by the exposure to 5 and 7.5 μM 7-KC (data not shown). The results revealed that the cell-killing effect of doxorubicin was significantly reduced by 7-KC in MCF-7 cells but not in MB-231 and MCF-7/ADR cells. Verapamil reversed the 7-KC-reduced doxorubicin cytotoxicity, suggesting the involvement of P-gp in the reduction by 7-KC in MCF-7 cells.

### Effects of oxysterols and ER antagonist on P-gp function in MCF-7, MB-231 and T-47D cells

**Figure 2:**
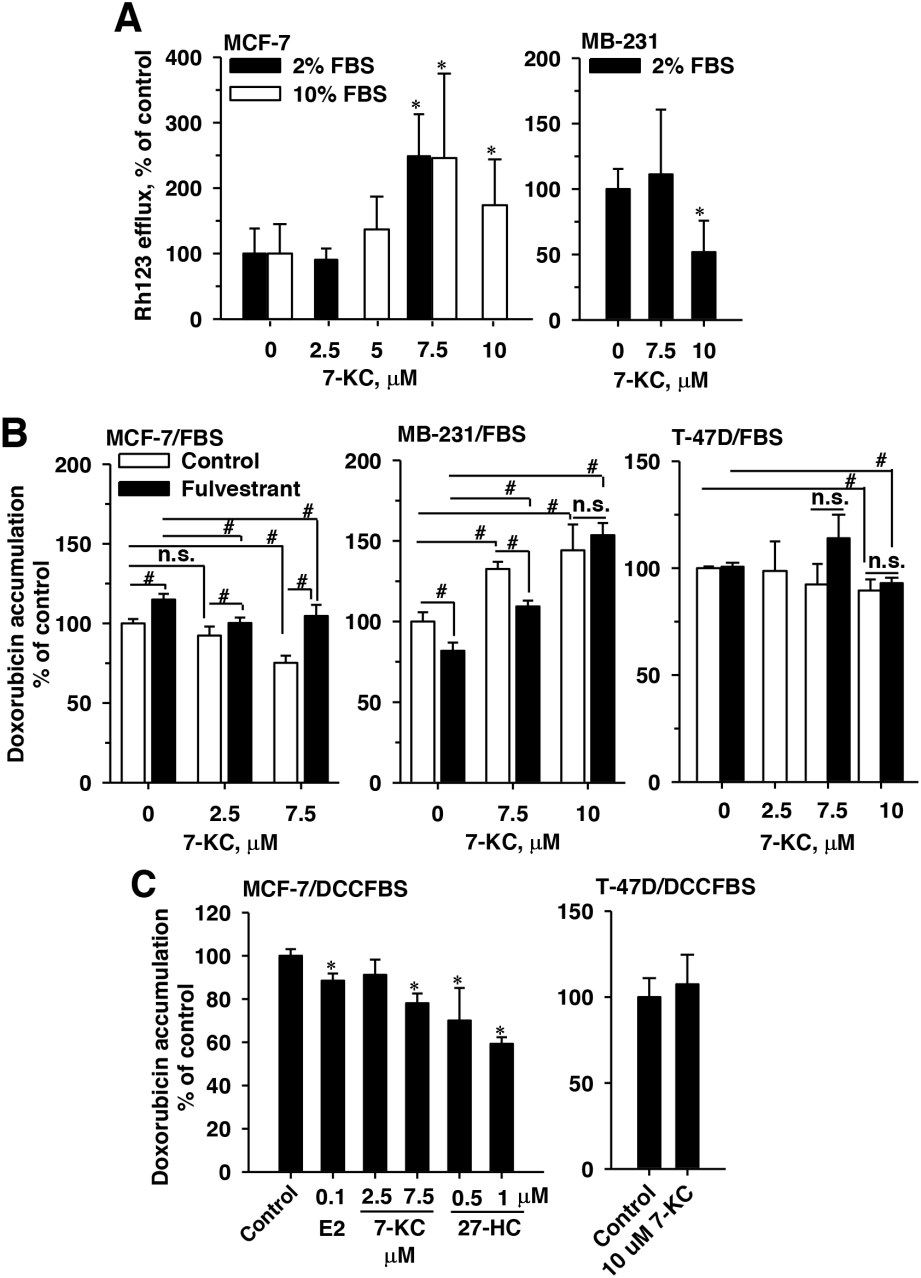
Effects of 7-ketocholesterol (7-KC), 27-hydroxycholesterol (27-HC), and fulvestrant on P-glycoprotein function in breast cancer cell lines P-glycoprotein function was monitored by verapamil inhibited efflux of rhodamine (Rh)123 **(A)** and cellular accumulation of doxorubicin **(B** and **C)**. Panel (A) shows the alterations of Rh123 efflux affected by 48-h exposure to 7-KC in cells cultured in 2% and 10% fetal-bovine-serum (FBS)-supplemented media. Rh123 efflux was determined as described in the Methods. Panel (B) shows the influence of the estrogen receptor (ER) antagonist fulvestrant on 7-KC-reduced doxorubicin accumulation in cells cultured in 2% FBS-supplemented medium. Cells were exposed to 5 μM fulvestrant together with 7-KC for 48 h and then doxorubicin accumulation was determined as described in the Methods. The results are presented as means ± SD of three independent experiments. To prevent the interference of trace E2 level in FBS-supplemented medium, MCF-7 or T-47D cells were exposed to estradiol (E2), 7-KC and 27-hydroxycholesterol (27-HC) in charcoal/dextran-stripped FBS (DCCFBS)-supplemented medium and doxorubicin accumulation were determined (C). Data are presented as means ± SD of three independent experiments. *p < 0.05, compared to the vehicle control. ^**#**^p < 0.05, the comparison between two treatments as indicated. n.s.: no significant differences between two treatments.

In MCF-7 cells cultured in a medium supplemented with 10% FBS, 48-h exposure to 7.5 and 10 μM 7-KC concentration-dependently stimulated the rhodamine 123 (Rh123) efflux function of P-gp by 65-148% (Figure 2A, left panel). When the FBS concentration was reduced to 2% in the medium, 7.5 μM 7-KC stimulated the efflux function by 150%. In contrast, in MB-231 cells, the efflux function was not affected by 7.5 μM 7-KC and was decreased by 10 μM 7-KC (Figure 2A, right panel). To link the 7-KC-decreased cytotoxicity of doxorubicin with the export function, cellular accumulation of doxorubicin (3 μM, 1 h) was determined in cells cultured in the medium supplemented with 2% FBS (Figure 2B). The exposure of cells to 3 μM doxorubicin for 1 h did not decrease cell growth (data not shown). Exposure of MCF-7 cells to 4β-HC, 7α-HC, 25-HC and 27-HC did not cause a significant change of doxorubicin accumulation (data not shown). Consistent with the reduced doxorubicin cytotoxicity in the MCF-7 cells (Figure 1C), 7.5 μM 7-KC caused a 25% decrease of cellular doxorubicin accumulation. In MB-231 cells, 7.5 and 10 μM 7-KC increased doxorubicin accumulation by 32% and 44%, respectively. In the ERβ predominant T-47D cells, 10 μM 7-KC caused a decrease of the mean doxorubicin accumulation by 10%.

To examine the involvement of ER in the change of doxorubicin accumulation, the effect of ER antagonist fulvestrant was examined. In MCF-7 cells cultured in the FBS-supplemented medium, the 7-KC-mediated decrease of doxorubicin accumulation was significantly eliminated by an ER antagonist fulvestrant to reach the vehicle control level (Figure 2B). Single exposure to fulvestrant increased cellular doxorubicin accumulation by 14%. Compared with fulvestrant treatment alone, less doxorubicin was retained in cells co-exposed to 7-KC and fulvestrant. In T-47D cells, fulvestrant did not affect the changes caused by 7-KC. Contrary to the response in MCF-7 cells, exposure of MB-231 cells to fulvestrant alone decreased doxorubicin accumulation by 29%. Compared with the single exposure to fulvestrant, 7.5 and 10 μM 7-KC exposures (together with fulvestrant) increased doxorubicin accumulation by 35% and 88%, respectively. The presence of fulvestrant significantly reversed the 7-KC-suppressed doxorubicin accumulation in MCF-7, but not in MB-231 and T-47D cells.

27-HC was present as a partial agonist and antagonist of ERα [27, 28]. The effects of E2, 7-KC and 27-HC on doxorubicin accumulation were determined in MCF-7 cells cultured in the DCCFBS-supplemented medium to eliminate the interference of E2 present in FBS. Cell growth was not affected by the replacement of FBS by DCCFBS (data not shown). In MCF-7 cells, E2, 7-KC and 27-HC significantly decreased cellular accumulation of doxorubicin when DCCFBS was used in the cell culture (Figure 2C). However, 7-KC exposure did not change the doxorubicin accumulation in T-47D cells when FBS was replaced by DCCFBS.

### The main distribution of 7-KC and 27-HC in the lipid raft domains in MCF-7 cells

**Figure 3:**
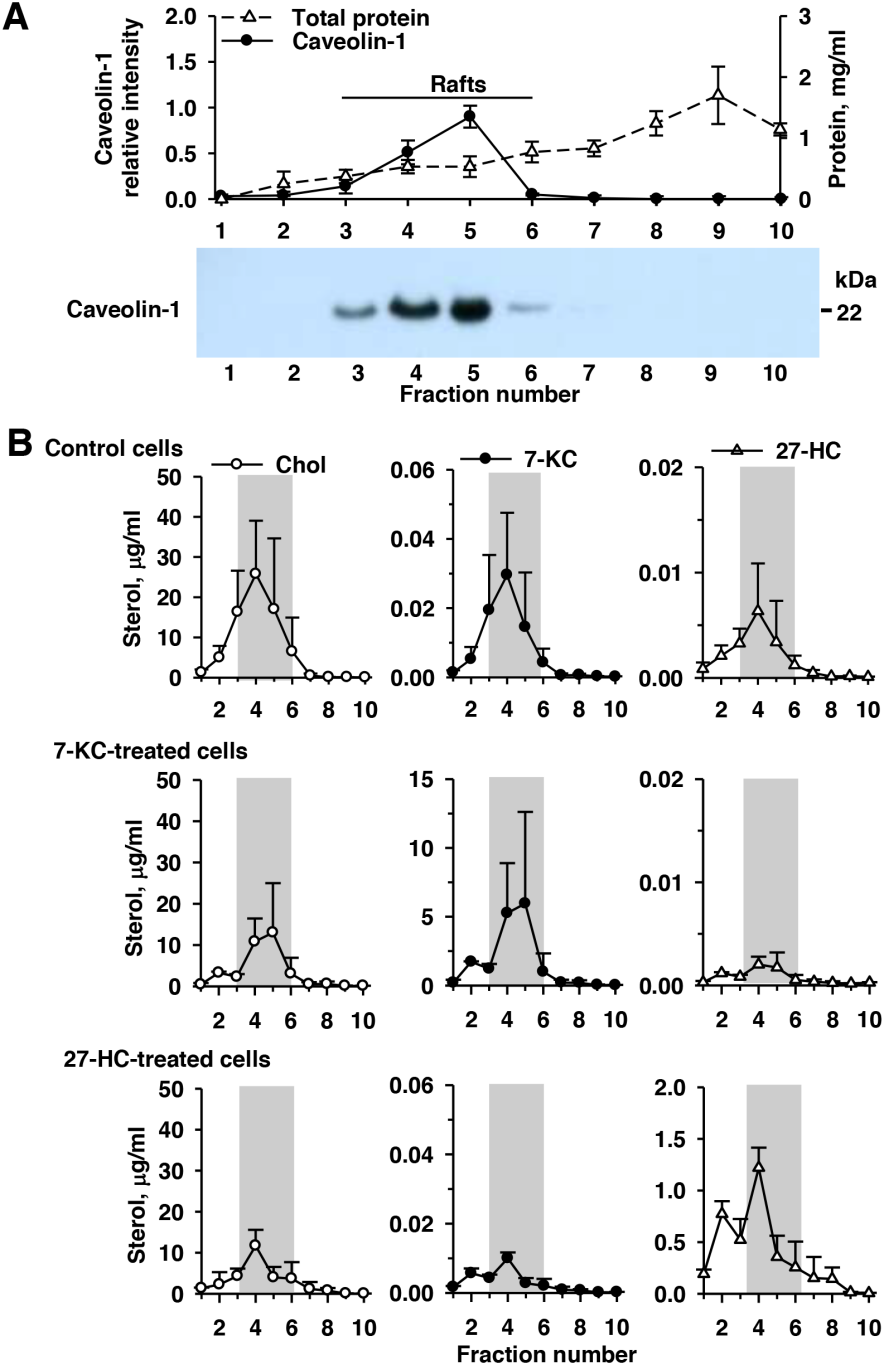
Accumulation of 7-ketocholesterol (7-KC) and 27-hydroxycholesterol (27-HC) in the lipid raft domains (gray bar) in the control, 7-KC-, and 27-HC-treated MCF-7 cells MCF-7 cells were exposed to 7.5 μM 7-KC and 2.5 μM 27-HC for 48 h. After fractionation of cell lysates on a sucrose density gradient, 10 fractions from the top (fraction 1, low density) to the bottom (fraction 10, high density) were collected. Panel **(A)** shows the results of the determination of protein concentration and immunoblotting analysis of caveolin-1 in each fraction. The level of caveolin-1 in fraction 5 was defined as 1.0. A representative blot (3.5 μg protein/well loaded) is shown on the bottom. The concentrations of cholesterol (Chol), 7-KC, and 27-HC in each fraction were measured **(B)**. The results are presented as means ± SD of three independent experiments.

When cells were cultured in the presence of 2% FBS, 7-KC—but not 27-HC—decreased doxorubicin accumulation in MCF-7 cells (Figure 2). Thus, membrane incorporation of 7-KC was examined in MCF-7 cells and comparison was made with 27-HC. Based on the respective non-toxic concentrations, MCF-7 cells were exposed to 7.5 μM 7-KC and 2.5 μM 27-HC for 48 h and sucrose gradient fractionations of cell lysates were collected after centrifugation. Determination of caveolin-1 and cholesterol contents in each fraction indicated that fractions 3–6 contained the lipid raft domains of the plasma membrane (Figure 3A). In the control cells, 7-KC and 27-HC appeared to be mainly distributed into fractions 3–6 (Figure 3B). Exposure to 7-KC and 27-HC did not elicit an alteration of their distribution into the lipid raft domains, indicating that their differential regulation of P-gp function was not due to distinct membrane distribution of 7-KC and 27-HC.

### Effects of 7-KC on the expression of P-gp in MCF-7and MB-231 cells

**Figure 4:**
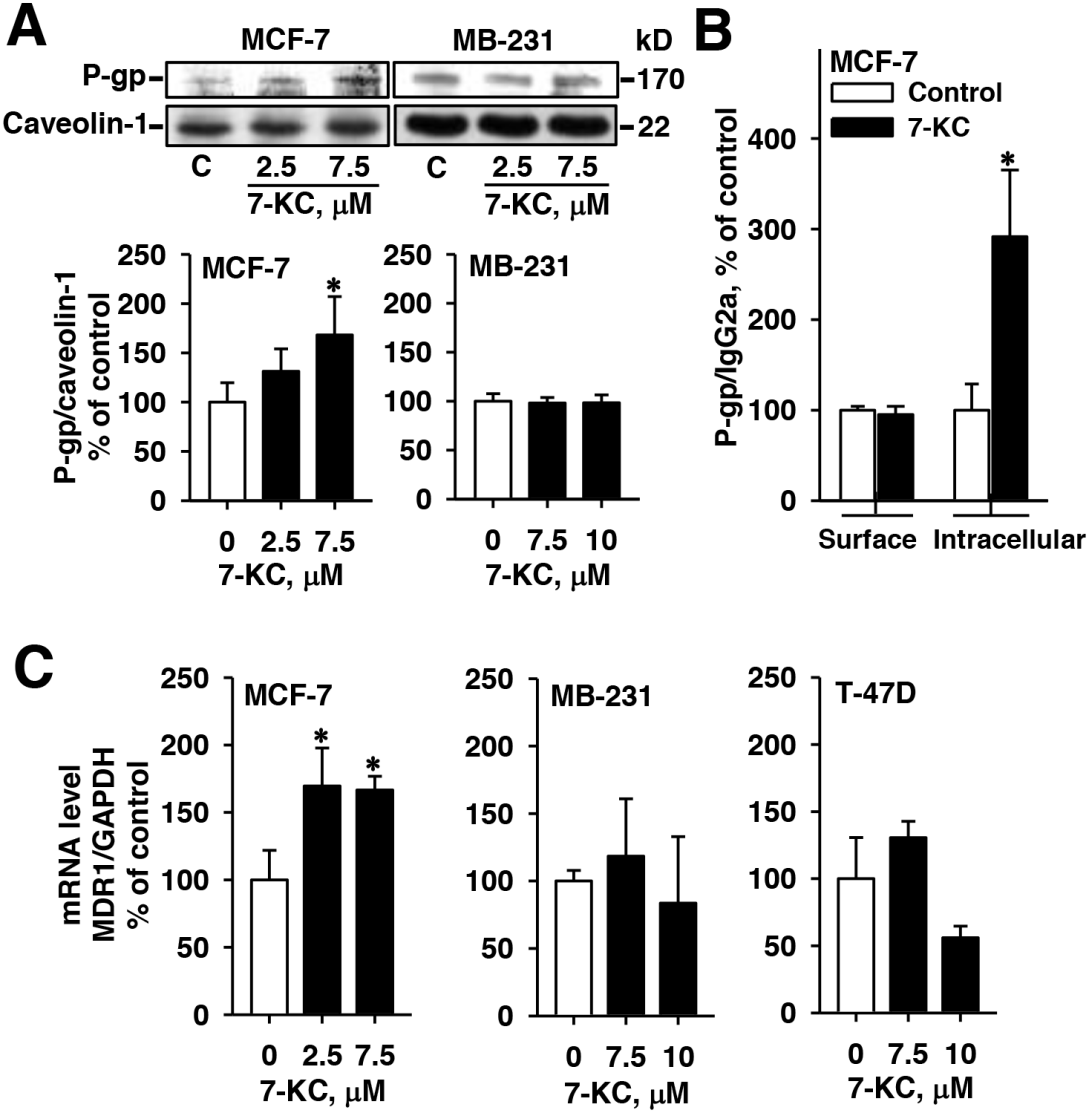
Alterations of protein and mRNA levels of P-glycoprotein (P-gp) in breast cancer cells after 48-h exposure to 7-ketocholesterol (7-KC) The levels of MDR1 gene-encoded P-gp protein was analyzed by immunoblotting analysis of crude membrane proteins (50 μg protein/well) **(A)** and by flow cytometry using PE-UIC2 antibody **(B)** as described in the Methods. The protein level of caveolin-1 was determined as the internal control. The band and fluorescence intensities of immune-reacted P-gp were normalized with the respective intensities of the internal controls, caveolin-1 and IgG2a. The results are presented as means ± SD of three and four independent experiments in the analyses of immunoblotting and flow cytometry, respectively. *p < 0.05. Panel **(C)** shows the changes of MDR1 mRNA analyzed by a real-time polymerase chain reaction. mRNA levels of MDR1 were normalized to the level of glyceraldehyde 3-phosphate dehydrogenase (GAPDH). The results are presented as means ± SD of three independent experiments. *p < 0.05, compared to the vehicle control.

Immunoblotting analysis revealed that exposure to 2.5 μM 7-KC did not significantly elevate the P-gp protein level. Exposure to 7.5 μM 7-KC stimulated the level of P-gp protein by 68% in MCF-7 cells (Figure 4A). By using the UIC2 antibody for the determination of cell surface P-gp, 48-h exposure to 7-KC did not increase the surface P-gp level. However, 7-KC significantly elevated the intracellular P-gp level 2-fold in MCF-7 cells (Figure 4B). Exposure to 2.5 and 7.5 μM 7-KC increased the mRNA levels of P-gp by 70% and 67% in MCF-7 cells, respectively (Figure 4C), indicating pre-translational upregulation of P-gp. In MB-231 cells, neither the mRNA nor the protein level of P-gp was affected by 7-KC-exposure. The MDR1 transcript was not affected by 7-KC in T-47D cells, either. The results showed that 7-KC stimulated the expression of P-gp at the pre-translational step in MCF-7 cells. The results of flow cytometry using the UIC2 antibody revealed that intracellular P-gp was induced.

### The influence of ERα knockdown, lipid raft integrity, N-acetyl cysteine and inhibitors of kinase signaling pathways on 7-KC-suppressed doxorubicin accumulation in MCF-7 cells

**Figure 5:**
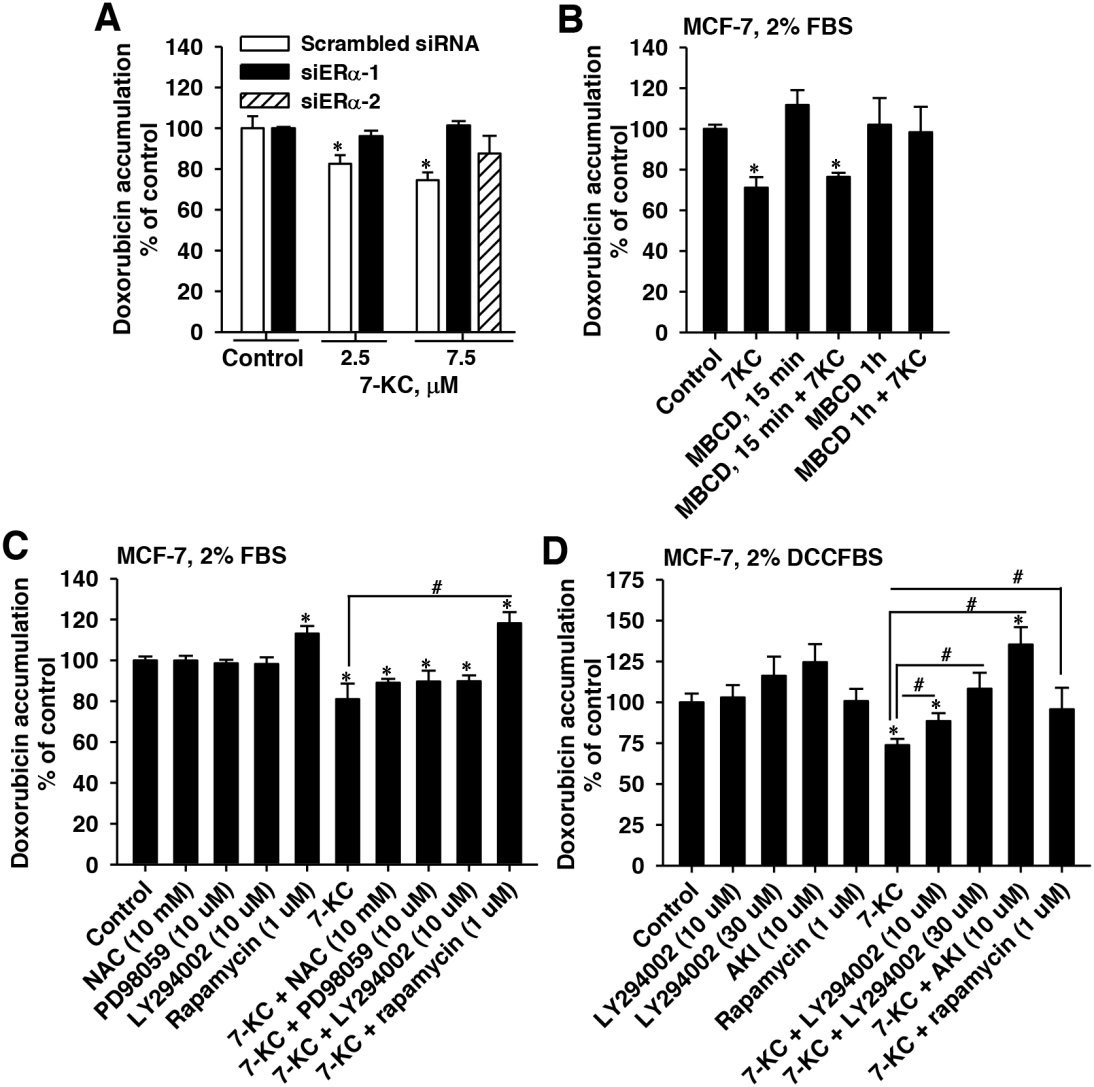
Influence of ERα knockdown, methyl-β-cyclodextrin (MBCD), N-acetyl cysteine (NAC), PD98059, LY294002, an Akt inhibitor (AKI), and rapamycin on 7-ketocholesterol (7-KC)-reduced doxorubicin accumulation in MCF-7 cells Panel **(A)** shows the 7-KC-induced changes of doxorubicin accumulation in ERα siRNA- and scramble-siRNA-transfected cells. The lipid raft integrity was disrupted using 10 mM MBCD. After siRNA-transfection or MBCD pre-exposure, cells were exposed to 7-KC in the medium supplemented with FBS for 48 h and then cellular doxorubicin accumulation were determined. The results are presented as means ± SD of three independent experiments. *p < 0.05, compared to the vehicle control. Panels **(C)** and **(D)** show the effects of NAC, PD98059, LY294002, AKI, and rapamycin on the doxorubicin accumulation in MCF-7 cells cultured in media supplemented with 2% FBS and charcoal-stripped FBS (DCCFBS), respectively. Cells were pre-exposed to a scavenger or inhibitor at a concentration as indicated in the figure for 1 h prior to 48-h co-treatment with scavenger/inhibitor and 7.5 μM 7-KC. After co-treatment, cellular accumulation (1 h) of doxorubicin was determined using 3 μM doxorubicin as described in the Methods. The results are presented as means ± SD of three to four independent experiments. *p < 0.05, compared to vehicle control. ^**#**^p < 0.05, the comparison between two treatments as indicated.

Because the 7-KC-mediated suppression of doxorubicin accumulation could be eliminated by fulvestrant in MCF-7 cells cultured in a medium supplemented with 2% FBS, the role of ERα was further examined by the knockdown of ERα using siRNA. After transfection of two different siERα, immunoblotting analysis showed that the expression level of ER protein was reduced by 72-86% (Supplementary Figure 2A). In ERα knockdown cells, 7-KC-exposure had no effect on cellular accumulation of doxorubicin (Figure 5A). The results indicated that ERα participated in the 7-KC-mediated suppression of doxorubicin accumulation.

To interrupt the lipid raft integrity, cells were exposed to 10 mM methyl-β-cyclodextrin (MBCD) before exposure to 7-KC in 2% FBS-supplemented medium. The MBCD exposure did not decrease cell growth. Exposure to MBCD at 37°C for 15, 30 and 60 min caused 34%, 48% and 52% reduction of cellular cholesterol level, respectively (Supplementary Figure 2B). In MCF-7 cells pre-exposed to MBCD for 1 h, 7-KC did not significantly change the doxorubicin accumulation (Figure 5B). The 7-KC-mediated reduction of doxorubicin accumulation showed dependence on the integrity of lipid rafts.

MCF-7 cells were exposed to a radical scavenger or signaling inhibitors for 1 h before 48-h co-exposure to 7-KC in 2%FBS or DCCFBS-supplemented medium. When FBS was used, N-acetyl cysteine alone had no effect on the doxorubicin accumulation and could not restore the doxorubicin accumulation decreased by 7-KC (Figure 5C). The 7-KC-reduced doxorubicin accumulation was not affected by the MEK (mitogen-activated protein kinase (MAPK)/extracellular signal-regulated kinase (ERK)) kinase inhibitor PD98059A. The nonselective PI3K inhibitor LY294002 (10 μM) had no effect either. However, the mTOR inhibitor rapamycin increased cellular doxorubicin accumulation in both the control and 7-KC-treated MCF-7 cells. To prevent the interference of E2, the FBS was replaced by DCCFBS. The involvement of PI3K/mTOR pathway was further examined. LY294002, Akt inhibitor (AKI), and rapamycin alone did not significantly change cellular doxorubicin accumulation (Figure 5D). However, LY294002 (10 and 30 μM) and AKI diminished the 7-KC-mediated decrease of doxorubicin accumulation. Rapamycin eliminated the decrease of doxorubicin accumulation by 7-KC. The PI3K/mTOR signaling pathway was crucial for the 7-KC-reduced doxorubicin accumulation in MCF-7 cells.

### Effects of E2, 7-KC and HCs on the doxorubicin accumulation and Trefoil factor 1 (TFF1) expression in MCF-7 and T-47D cells

**Figure 6:**
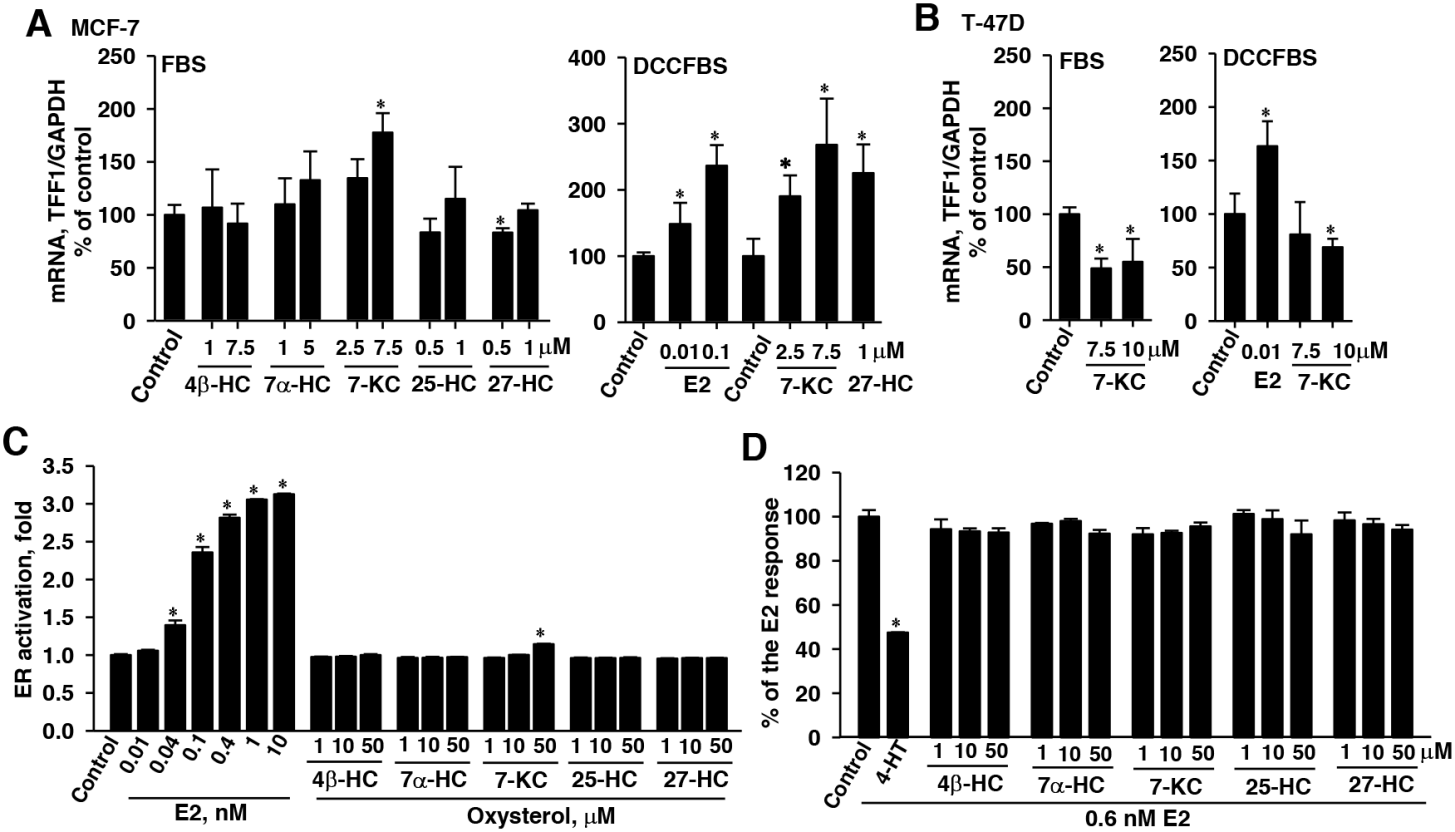
Effects of 7-ketocholesterol (7-KC) and hydroxycholesterols (HCs) on mRNA levels of the ERα target TFF1 gene in MCF-7 **(A)** and T-47D **(B)** cells and the estrogen receptor (ER) agonistic **(C)** and antagonistic **(D)** effects of oxysterols in a yeast reporter assay system. Breast cancer cell lines were cultured in a medium supplemented with either FBS or DCCFBS. Cells were exposed to 7-KC and HCs for 48 h and the induction of TFF1 by 48-h exposure to estradiol (E2) in DCCFBS-supplemented medium was performed as the positive control. The results show the mRNA levels of TFF1 normalized to glyceraldehyde 3-phosphate dehydrogenase (GAPDH) mRNA. The results are presented as means ± SD of three independent experiments with two determinations in each experiments. *p < 0.05, compared to the vehicle control. In panels (C) and (D), the ER activation by oxysterols was assayed in a yeast system transfected with a LacZ reporter construct carrying the response element for ER complex (YES assay). Yeast cultures were exposed to estradiol (E2) and oxysterols for 72 h. The results are presented as means ± SD of three separate determinations. The antagonistic effects of 4-hydroxytamoxifen (4-HT) and oxysterols (72 h) on the E2-stimulated ER activation were determined using 0.6 nM E2 in the YES assay. The results are presented as means ± SD of three separate determinations. *p < 0.05, compared to the vehicle control.

Because ERα was shown to be crucial for the P-gp induction by 7-KC (Figure 6) and 27-HC has been reported to act as a partial ER agonist/antagonist [5, 29], mRNA levels of a ER target TFF1 were determined in oxysterol-exposed cells cultured in FBS and DCCFBS-supplemented media. In the culture system containing FBS, the level of TFF1 mRNA was significantly elevated by the exposure to 7.5 μM 7-KC in MCF-7 cells, whereas 4β-, 7α-, and 25-HC had no effects (Figure 6A, left panel). The TFF1 mRNA level was decreased by 0.5 μM 27-HC. The effects of E2, 7-KC, and 27-HC on TFF1 expression were further examined in MCF-7 cells cultured in DCCFBS-supplemented medium. E2 (at 0.01 and 0.1 μM) caused 48% and 136% increases of TFF1 mRNA levels, respectively (Figure 6A, right panel). Both 7-KC and 27-HC stimulated the expression of TFF1.

In T-47 D cells, cultured in the medium supplemented with FBS, 7.5 and 10 μM 7-KC decreased the mRNA level of an ER target gene TFF1 by 51% and 45% in T-47D cells, respectively (Figure 6B, left panel). When the culture medium was supplemented with DCCFBS (Figure 6B, right panel), E2 stimulated the expression of TFF1 transcript by 63%, whereas 7-KC did not change the TFF1 transcript level. The results revealed that 7-KC and 27-HC were capable of inducing TFF1 expression in MCF-7 cells and the induction by 27-HC could be suppressed by charcoal/dextran-removable serum factors. In T-47D cells, E2, but not 7-KC induced TFF1 expression.

### The ERα agonistic and antagonistic effects of oxysterols in a YES assay

To determine the ERα agonistic and antagonistic effects of oxysterols, a YES assay using a yeast system for the determination of ERα-mediated reporter gene expression was studied. E2 stimulated the expression of reporter in a dose-dependent manner and caused a maximal 3-fold increase after 72 hours (Figure 6C). In this system, ERα-activated transcription activity was stimulated by 14% after exposure to 7-KC at a concentration as high as 50 μM. However, the other oxysterols including 27-HC did not show the ERα agonistic effect in this reporter assay system. In the determination of ERα antagonistic effects, the known ER antagonist 4-HT (20 μM) caused a 52% decrease in the ER activation elicited by 0.6 nM E2 (Figure 6D). None of the oxysterols showed ERα antagonistic activity at concentrations up to 50 μM. Results of reporter assay indicated that 7-KC was a weak ERα activator. HCs did not show agonist/antagonist effect in this reporter system.

## DISCUSSION

Oxysterols have been reported to be a risk factor for tumor promotion in breast cancer, in which the modulation of estrogenic activity is proven to be important [5]. 27-HC stimulated mammary tumor volume and the metastasis of mammary cancer to the lungs in ovariectomized, immunocompromised mice carrying the mammary tumor virus-polyoma middle T-antigen [5]. Overnight treatment with 27-HC at 10 μM has been reported to stimulate ER transcriptional activity by ∼30-fold in human cervix adenocarcinoma HeLa cells transfected with an expression construct of ERα, in which E2 caused an activation > 60-fold induction [28]. However, in another ER reporter assay in ERα-transfected HeLa cells, 24-h exposure to 7-HC, 7-KC, 25-HC, or 27-HC at 10 μM did not elicit significant activation of ER transcriptional activity, whereas E2 caused a 10-fold induction [29]. As shown by the YES assay, commonly used for the examination of ER agonistic and antagonistic activities, 0.04-10 nM E2 stimulated the expression of the reporter gene by up to 3-fold (Figure 6C). The induction fold of reporter stimulation by E2 in our findings was consistent with results in the other reports using this YES assay [30, 31]. The HCs, including 27-HC, did not activate the reporter gene expression in the YES assay. The differences in the results obtained from different reporter assays may be due to the distinct sensitivities of ER activation assays, potentially resulting from factors, such as the number of repeats of responsive element in the reporter construct and cell types. Although the ER activation by 7-KC was relatively weak in the YES assay and required a very high concentration (50 μM) of 7-KC (Figure 6C), we discovered that 7-KC was capable of ERα activation. Depending on the extracellular E2 level, promoter context and cell type, oxysterols, such as 27-HC, have been recognized as an endogenous selective ER modulator (SERM) and may exert partial ER agonistic and antagonistic properties [5, 27–29, 32]. When MCF-7 cells were cultured in DCCFBS-supplemented medium, our findings revealed that both 7-KC and 27-HC stimulated the expression of ER-target gene TFF1. Although the antagonistic activity of 27-HC was below the detection threshold in the YES assay (Figure 6D), 27-HC did not induce TFF1 expression when the oxysterol exposure was performed in the medium supplemented with FBS, suggesting the interference of a low concentration of E2 in the 2% FBS-supplemented medium. The results of TFF1 induction and ERα-activated reporter expression reveal that 7-KC and 27-HC exert estrogenic activity and charcoal/dextran removable serum factors, such as E2 may suppress the estrogenic properties of 27-HC (Figure 6A and 6B). The differential stimulation of TFF1 by 7-KC and 27-HC (Figure 6A) was consistent with their effects on the doxorubicin accumulation (Figure 2B and 2C) in MCF-7 cells cultured in the medium supplemented with either FBS or DCCFBS.

Increased drug efflux activities of ABC transporters appear to be one of the primary causes for the resistance of cancer cells to cytostatics, such as doxorubicin [8, 9]. The mean plasma concentration of doxorubicin in overweight patients was about half of that in patients of normal weight when doxorubicin was administrated based on body surface area [26]. Following exposure to 0.1, 1 and 3 μM doxorubicin, 7-KC decreased the cytotoxic effect of doxorubicin in MCF-7 (Figure 1). 4-, 7- and 27-HC enhanced doxorubicin cytotoxicity without affecting doxorubicin accumulation, indicating that the enhanced cytotoxicity could not be linked to the change of intracellular doxorubicin accumulation. Other factors including synergistic cytotoxic effects might be involved in it. The 7-KC-increased doxorubicin resistance could be suppressed by a P-gp inhibitor, verapamil, but not by inhibitors of MRP and BCRP. Consistent with the decrease of intracellular accumulation of doxorubicin, the Rh123 efflux function of P-gp was stimulated by 7-KC in the ERα-predominant MCF-7 cell line but not in ERα(-)MB-231 cell line. In MCF-7 cells, 7-KC induced the expression level of P-gp, mainly intracellular P-gp (Figure 4). The increased intracellular P-gp has been suggested to increase doxorubicin resistance potentially through the change of intracellular drug export across membranes of cellular organelles [33]. 7-KC can be one of the risk factors for the increased resistance to the doxorubicin therapy against breast cancer. Further animal study should be done to illustrate the effect of 7-KC on doxorubicin sensitivity *in vivo*. On the other hand, T47D cells was resistant to the 7-KC-mediated P-gp induction. 7-KC induced P-gp and decreased doxorubicin accumulation in human hepatoma Huh-7 and HepG2, but not in the immortalized primary human hepatocytes HuS-E/2 cells [22]. 7-KC mediated P-gp induction showed dependence on cell types.

The exposure to fulvestrant and ERα knockdown restored the doxorubicin accumulation suppressed by 7-KC in MCF-7 cells (Figures 2B and 5A). The single exposure to fulvestrant increased doxorubicin accumulation, probably due to the inhibition of basal ER activation or competitive inhibition of P-gp function [34]. Our findings revealed that a functional ERα was important for the P-gp induction by 7-KC in MCF-7 cells. 7-KC reduced doxorubicin accumulation in MCF-7 cells cultured in either 2% FBS- or DCCFBS-supplemented medium (Figure 2B and 2C). However, in the ERβ-predominant T-47D cells, doxorubicin accumulation remained unchanged after exposure to 7-KC in the DCCFBS-supplemented medium (Figure 2C). Shi et al. [35] reported that overexpression of ERα upregulated the MDR1 mRNA level in ERα(+) MCF-7 and SR-75-1 cells. E2 (100 nM) stimulated the expression of MDR1 mRNA without significantly affecting the MRP1 and BCRP mRNA levels in the primary culture of human trophoplasts [36]. E2 exposure increased doxorubicin resistance in MCF-7 but not in T-47D cells [33]. These results together with our findings reveal that ERα signaling can be crucial in the P-gp upregulation. However, in the P-gp overexpressed MCF-7/ADR cells, exposure to 7-KC did not decrease the accumulation of doxorubicin. Compared to 10 pM E2-treated cells, cellular Rh123 accumulation was similar to that in 100 pM E2-treated MCF-7 cells but lower than that in 100 pM E2-treated MCF-7 cells/MDR cells [37]. Exposure of resistant MCF-7/MDR cells to E2 downregulated the P-gp protein level. In the sensitive and resistant cells, E2 may elicit distinct signaling and the differential P-gp modulatory effect of 7-KC needs further investigation. Tumor ER status and extracellular E2 levels can be important for the estrogenic activities of oxysterols, associated with the P-gp induction. 7-KC can be one of the risk factors for the poor therapeutic outcome of doxorubicin in breast cancer patients carrying a high expression ratio of ERα/ERβ.

Several signaling factors, e.g. MAPKs and mTOR, have been noted in the induction of P-gp [22, 38]. Our previous study of hepatoma Huh-7 cells revealed that 7-KC distributed mainly in the non-lipid raft domains in 7-KC-treated cells and PI3K/mTOR signaling is crucial for the post-transcriptional induction of P-gp by 7-KC, whereas the oxidative stress made a minimal contribution [22]. In contrast to the distribution in Huh-7 cells, both 7-KC and 27-HC were mainly distributed in the lipid raft domains of MCF-7 cells with or without exposure to oxysterols (Figure 3). The incorporation of 7-KC into the lipid raft domain showed differences in different cell types. The increased ER translocation to the lipid raft domain induced kinase signaling in breast cancer cells [39]. Disruption of lipid rafts using MBCD eliminated E2-induced signaling in human platelets [40], revealing the necessity of lipid raft integrity. In MCF-7 cells, MBCD also eliminated the 7-KC-mediated reduction of doxorubicin accumulation (Figure 5B), indicating the importance of the integrity of lipid rafts. Like hepatoma cells, oxidative stress and MEK contributed minimally to 7-KC-mediated upregulation of P-gp function in MCF-7 cells. ER activation could stimulate mTOR signaling [41], and mTOR was found to co-localize with ERα upon estrogen stimulation in MCF-7 cells [42]. Because the mTOR inhibitor rapamycin increased doxorubicin accumulation in both vehicle- and 7-KC-treated MCF-7 cells when FBS was used in the culture (Figure 5C), the influence of rapamycin was further examined using DCCFBS. The decrease of doxorubicin accumulation was significantly reversed by inhibitors of PI3K, Akt and mTOR (Figure 5D). Thus, the mTOR activation through PI3K/Akt pathway can be involved in the induction of P-gp by 7-KC in MCF-7 cells. The mTOR inhibitors have shown to improve the outcome of taxane and tamoxifen treatment in patients with advanced and ER (+)breast cancer [43]. Our findings suggested that 7-KC- and 27-HC-reduced doxorubicin sensitivity could also be the therapeutic target for mTOR inhibitors.

In summary, our findings demonstrate that ERα makes a major contribution to the 7-KC-mediated pre-translational up-regulation of P-gp, leading to a decrease of intracellular doxorubicin accumulation and cell-killing effect of doxorubicin in breast cancer cells with a high ERα/ERβ expression ratio. Although 7-KC weakly activated ERα in the YES assay, 7-KC stimulated the expression of TFF1 in MCF-7 cells (Figure 6A and 6C). Following removal of E2 present in FBS using charcoal/dextran, both 7-KC and 27-HC induced TFF1 transcript and suppressed doxorubicin accumulation. These results indicate that the decreased doxorubicin accumulation through the induction of P-gp is related to the estrogenic activities of oxysterols. The relative levels of oxysterols and the status of menopause can be important for the adjustment of dosing regimen of doxorubicin in the neoadjuvant and adjuvant chemotherapy of breast cancer patients with a high ratio of ERα/ERβ. The reduction in the level of circulating cholesterol and its oxidation to form 27-HC and 7-KC may be useful strategies in the chemotherapeutic sensitization. Further studies in mice bearing breast cancer and the determinations of oxysterol profiles in plasma and tissue samples from breast cancer patients are important to elucidate the association of oxysterols with the outcomes of chemotherapy.

## MATERIALS AND METHODS

### Chemicals, solvents and serum

*N*-acetyl cysteine, AKI, dextran-coated charcoal (DCC, charcoal/dextran), doxorubicin hydrochloride, E2, fulvestrant, fumitremorgin C, 4-hydroxytamoxifen (4-HT), 25-HC, indomethacin, 7-KC, LY294002, 3-(4, 5-dimethyl-thiazol-2yl)-2, 5-diphenyl tetrazolium bromide (MTT), MBCD, PD98059, rapamycin, Rh123 and verapamil were purchased from Sigma-Aldrich (St. Louis, MO, USA). 4β- and 7α-HC were purchased from Steraloids (Newport, RI, USA). 27-HC was purchased from Avanti Polar Lipids (Alabaster, AL, USA). Dichloromethane, ethanol, methanol, sodium chloride, sucrose, and Triton X-100 were purchased from Merck KGaA (Darmstadt, Germany). A protease inhibitor cocktail (Complete tablet, EDTA-free) was purchased from Roche Diagnosics (Mannheim, Germany). FBS was purchased from Biological Industries Israel Beit Haemek (Kibbutz Beit Haemek, Israel).

### Cell culture and exposure

MCF-7, T-47D and MB-231 cells were purchased from Bioresource Collection and Research Center, Food Industry Research and Development Institute, Hsinchu, Taiwan. The characterization of cell lines was done by the same center using STR polymerase chain reaction (PCR) (AmpFLSTR Identifier PCR amplification kit, Applied Biosystems/Thermo Fisher, Foster City, CA, USA) DNA analysis. Genomic DNA was isolated (PureLinkTM genomic DNA mini kit, Invitrogen, Carlsbad, CA, USA) and DNA profiling was analyzed using ABI 3730 Sequencer (Applied Biosystems Inc., Foster City, CA, USA). MCF-7/ADR cells were prepared by stepwise exposure of MCF-7 cells to an increasing concentration of doxorubicin [44]. Cells (MCF-7 and MB-231: within 30 passages; T-47D: within 20 passages; MCF-7/ADR: within 15 passages) were cultured in Dulbecco’s Modified Eagle medium (DMEM) supplemented with 10% (v/v) fetal bovine serum (FBS), 1% (v/v) of the nonessential amino acid mixture (100 ×), 2 mM L-glutamine and 1% (v/v) of the penicillin/streptomycin/amphotericin mixture (100 ×) under a humidified atmosphere at 37°C with 5% CO_2_. After overnight culture following seeding, cells were washed with phosphate-buffered saline (PBS) containing 150 mM NaCl, 2.7 mM KCl, 1.3 mM KH_2_PO_4_, and 8.1 mM Na_2_HPO_4_ (pH 7.4). Cells were then exposed to oxysterols (stock solution in ethanol) in the medium supplemented with 2% FBS (v/v). To examine the role of estrogenic activity and the influence of E2, the cells were exposed to oxysterols or 1–100 nM E2 (stock solution in methanol) in a medium supplemented with 2% (v/v) charcoal/dextran-stripped FBS (DCCFBS). FBS was heat-inactivated (56^°^C, 30 min) and stored in aliquots at -20^°^C before use. DCCFBS was prepared by gently mixing 100 ml of heat-inactivated FBS with 2 g DCC at 4^°^C overnight. After centrifugation at 3000 g for 15 min at 4^°^C, the supernatant was filtered through a sterile filter (0.2 μm, Thermo Fisher Scientific, Rochester, NY, USA) and the filtrate was used as the DCCFBS. The concentration of E2 in DCCFBS was determined using an E2 EIA kit (Cayman Chemical Co., Ann Arbor, MI, USA). Of the E2 present in FBS, 96% was removed. The concentration of E2 in the DCCFBS-supplemented medium was < 1 pM. The control cells were exposed to the same concentration of vehicle in the medium, and the final concentration of vehicle in all treatments was ≤ 0.2%. The growth of viable cells was monitored by trypan blue exclusion and 3-(4, 5-dimethyl-thiazol-2yl)-2, 5-diphenyl tetrazolium bromide (MTT) reduction assays as previously described [22]. Cells at 70-80% confluence were exposed to oxysterols at a concentration below their respective cytotoxic concentrations in the following studies.

### The efflux function of P-glycoprotein and accumulation of doxorubicin

The P-gp transporter-mediated efflux function was determined by measuring the retention of Rh123 in cells as previously described [22]. Primarily, cells were seeded (2 × 10^5^ cells/well) on a 12-well plate for 24 h and then incubated with 5 μM Rh123 in medium at 37^°^C in a CO_2_ incubator for 1 h. After two washes with ice-cold PBS in the dark, cells were incubated with Rh123-free medium in the absence and presence of 30 μM verapamil for 3 h. After three washes with ice-cold PBS, the fluorescence of Rh123 retained in cells was measured using a microplate reader (SpectraMax M5, Molecular Devices, Sunnyvale, CA, USA). The decrease of export of Rh123 in the presence of verapamil was determined to monitor the efflux function of P-gp. To determine the accumulation of doxorubicin, cells were seeded (5 x10^5^ cells/well) on a 6-well plate for 24 h and then exposed to oxysterols or E2 in either FBS- or DCCFBS-supplemented medium for 48 h. Subsequently, cells were washed with PBS twice, treated with trypsin/EDTA (Biological Industries, Kibbutz Beit HaeMek, Israel) and neutralized with medium. After centrifugation at 200 g for 5 min at room temperature, cell pellets were resuspended in the medium containing 3 μM doxorubicin and incubated for 1 h. The exposure concentration of 3 μM was in the linear range of the fluorescence of cellular doxorubicin versus the doxorubicin exposure concentrations (data not shown). After washing with ice-cold PBS, the mean fluorescence of doxorubicin retained in 2 × 10^4^ cells was measured using a flow FACSCalibur cytometer (excitation: 480 nm; emission: 564–660 nm; BD Biosciences, San Jose, CA, USA).

### Isolation of lipid rafts and quantification of 7-KC, 27-HC and cholesterol

Lipid rafts were isolated from MCF-7 cells using a method modified from the report of Royer et al. [45]. In a dish (10 cm i.d.), cells at 70-80% confluence were exposed to oxysterols. After 48 h, cell lysate was prepared from 6 × 10^7^ cells and subjected to sucrose gradient centrifugation as previously described [22]. After 24-h centrifugation at 36,000 rpm (4°C) in a SW41 rotor (Beckman Coulter, Fullerton, CA, USA), each fraction of 1.1 ml was collected from the top to the bottom of the tubes, mixed, and stored at -20°C. An aliquot (1 ml) of each fraction was extracted with 2 ml dichloromethane, and 1 ml of the bottom layer was evaporated to dryness under an N_2_ stream and subjected to the liquid chromatography (LC)-mass spectrometry (MS, with atmospheric pressure chemical ionization (APCI)) analysis as previously described [22]. Briefly, dry lipid rafts were dissolved into 40 μl of CH_3_OH/CH_3_CN (1/3) with stable isotope-labeled d_6_-27-HC, d_7_-7-KC, and d_7_-cholesterol (10 ng each) added as internal standards. Oxysterols were resolved by UPLC (50°C) on an Acquity BEH octadecylsilane (C18) column (1.7 μm; 1 mm × 100 mm) (Waters) using solvent mixtures of 25% H_2_O/CH_3_CN to 10% 2-propanol/CH_3_CN over 6 min with a linear gradient at a flow rate of 0.16 ml/min and quantified by positive APCI/MS/MS-SRM monitoring of product ions: *m/z* 161.2 (27-HC), *m/z* 383.2 (7-KC), *m/z* 161.2 (cholesterol) originating from *m/z* 385.2 (27-HC), *m/z* 401.2 (7-KC), *m/z* 369.2 (cholesterol), respectively, and *m/z* 161.2 (d_6_-27-HC), *m/z* 390.2 (d_7_-7-KC), *m/z* 161.2 (d_7_-cholesterol) originating from *m/z* 381.2 (d_6_-27-HC), *m/z* 408.2 (d_7_-7-KC), and *m/z* 376.2 (d_7_-cholestol), respectively. Quantification was done using isotope ratios and internal standard curves. An aliquot (2 μl) of each fraction was subjected to protein concentration determination. Immunoblotting analysis of caveolin-1 in fractions was performed as described below. In the MBCD treated cells, cellular free cholesterol was determined using a Cholesterol Quantitation Kit (Sigma-Aldrich, St. Louis, MO, USA).

### Immunoblotting analysis of P-glycoprotein

Cells were collected from the dishes using a cell lifter and then washed twice with PBS. Whole-cell lysate was prepared using a hypotonic buffer, and crude membranes were collected after centrifugation following the method reported by König et al. [46]. Protein concentrations of crude membranes were estimated by a dye-binding assay following the instruction manual for the Bio-Rad Protein assay kit (Bio-Rad, Hercules, CA, USA). In the determination of P-gp (170 kD) and caveolin-1 (22 kD), crude membrane proteins (50 μg) were subjected to sodium dodecyl sulfate-polyacrylamide gel electrophoresis (SDS-PAGE) using a stepwise gradient polyacrylamide gel (3.5% (w/v) stacking gel and 7.5% (upper zone) and 10% (bottom zone) separation gel. Electrophoresis was carried out using the discontinuous system reported by Laemmli [47]. Following electrophoresis, proteins were transferred from the slab gel to a nitrocellulose membrane following the method reported by Towbin et al. [48]. Mouse monoclonal antibodies against P-gp (P 7965) were purchased from Sigma-Aldrich (St. Louis, MO, USA). P7965 does not recognize human MDR3 and mouse mdr1a and mdr3 [49, 50]. Rabbit polyclonal anti-caveolin-1, which immunoreacted with human, mouse and rat caveolin-1, was purchased from BD Biosciences Pharmingen (Franklin Lakes, NJ, USA). Anti-P-gp (1:500) and anti-caveolin-1 (1:2000) were diluted using PBS containing 1% non-fat milk (w/v). Antibody incubation was carried out at 4^°^C overnight, and non-selective binding was reduced by 4 washes with PBS containing 0.5% Tween 20 (v/v). Immunoreactive proteins were detected by horseradish peroxidase-conjugated secondary antibodies (1:1000) (goat anti-mouse and anti-rabbit IgGs, Thermo Fisher Sci., Wilmington, DE, USA). The bands were visualized using chemiluminescence kits ECL Select (PRN2235, high sensitivity) and ECL (PRN2106) (Amersham, GE Healthcare Life Sci., Pittsburgh, PA, USA) for the detection of caveolin-1 and the other proteins, respectively. Protein band intensity was analyzed using the image-processing program ImageJ (Rasband, W.S., ImageJ, MD, USA).

### Immunofluorescence detection of P-glycoprotein level by flow cytometry

To determine the expression levels of cell surface and intracellular P-gp, monoclonal antibody UIC2 was used [33, 51]. In the determination of cell surface proteins, cells were collected, immunostained with phycoerythrin (PE)-labeled UIC2 (ab93590, Abcam, Cambridge, MA, USA) and analyzed using the flow cytometric determination. In the determination of intracellular proteins, cells were seeded (3 × 10^6^ cell on dish (15 cm i.d.), 48 h) and then collected and permeabilized using PBS containing 0.2% bovine serum albumin (BSA)(w/v) and 0.1% saponin (w/v) (at room temperature for 5 min). After centrifugation at 400 g for 5 min at room temperature, cells were resuspended in 100 μl of PE-UIC2 (1:100) in the permeabilization buffer and incubated at room temperature for 30 min in the dark. Cells were washed with 200 μl of cold PBS containing 0.2% BSA (w/v) and centrifuged at 400 g for 5 min. Fluorescent isotype control immunoglobulin G, PE-IgG2 (ab91363, Abcam, Cambridge, MA, USA) was used for immunostaining as the internal control. Cells were resuspended in 0.5 ml ice-cold PBS and the mean fluorescence intensity (2 × 10^4^ cells) was determined using FACSCalibur cytometer and FACS/Cell Quest software (BD Biosciences, San Jose, CA, USA).

### Semi-quantitative reverse transcription (RT)-polymerase chain reaction (PCR) analysis of the MDR1 and TFF1 mRNA

Cellular total RNA was isolated using TRIsure reagent (Bioline Reagents., London, UK) following instructions of the supplier. Total RNA samples (5 μg, *A*_260_/*A*_280_:1.8-2.1) (NanoDrop 1000, Thermo Fisher Scientific, Wilmington, DE, USA) were subjected to reverse transcription using RevertAid First Strand cDNA synthesis Kit (Thermo Fisher Scientific) in a final volume of 20 μl. The RT step occurred at 42^°^C for 60 min and then at 70^°^C for 5 min. The resulting cDNA (1 μl RT product) was subjected to PCR amplification using Bioline SYBR No-ROX kit (Bioline Reagents, London, UK). The primer sets used for amplification of MDR1 were described previously [22]. The other sets were glyceraldehyde 3-phosphate dehydrogenase (GAPDH) (NM_002046.5; amplicon: 307 b.p.): forward, CGGAGTCAACGGATTTGGTCGTAT; reverse, AGCCTTCTCCATGGTGGTGAAGAC, and TFF1 (NM_003225.2; amplicon: 105 b.p.): forward, CATCGACGTCCCTCCAGAAGAG; reverse, CTCTGGGACTAATCACCGTGCTG [52]. In the amplification of MDR1, TFF1, and GAPDH, an initial enzyme activation step of 95^°^C for 2 min was followed by 40 cycles each at 95^°^C for 5 s, 60^°^C for 15 s and 72^°^C for 10 s. The amount of target cDNA in each sample was estimated by determining a fractional PCR threshold cycle number (*C*_t_ value) using SYBR green staining and LightCycler 480II (Software version 1.5.0.39; Roche Diagnostics GmbH, Mannheim, Germany). Data were analyzed with dynamic integration time mode. The *C*_t_ value and log μg of total RNA showed linear relationship (r^2^ = 0.99∼1.00) and the intra-assay variation (four determinations) was <5%. The relative mRNA expression levels were normalized to the GAPDH transcript level, which allowed the target cDNA calculation by 2^-(Ct^ ^*MDR1 or TFF1*^ ^–Ct^ ^*GAPDH*)^.

### Transfection of estrogen receptor siRNA

MCF-7 cells (5 × 10^4^ cells/well) were seeded in a 6-well plate and grown in an incubator. At 24 h after seeding, cells were transfected with ERα siRNA (50 nM) and scrambled siRNA (40 nM) using lipofectamine 2000 (Invitrogen, Carlsbad, CA, USA) following the supplier’s instructions. Human ER siRNA (s4823 and s4824) and scrambled siRNA were purchased from Ambion (Life Technologies, Thermo Fisher Scientific). s4823, sense: ACAUCAUCGGUUCCGCAtt, antisense: UGCGGAACCGAGAUGAUGUag; s4824, sense: CAGGCACAUGAGUAACAAAtt, antisense: UUUGUUACUCAUGUGCCUGat. Cells were then incubated at 37^°^C for 6 h. After removal of the transfection medium, cells were cultured in a medium supplemented with 10% FBS (v/v) for 18 h. Transfected cells were exposed to ethanol or 7-KC in 2% (v/v) FBS-containing medium for 48 h and the accumulation of doxorubicin was determined. The expression level of ERα protein was determined using immunoblotting analysis as described above to ensure the knockdown of ERα protein (66 kD). Primarily, crude membrane proteins (30 μg/well) were separated using a 7.5% (w/v) polyacrylamide gel and transferred to nitrocellulose membranes. Mouse monoclonal antibodies against ERα (SC-8005) (D-12, raised against 2–185 amino acids at N-terminal) was purchased from Santa Cruz (Dallas, TX, USA). SC-8005 did not recognize human Erβ [53]. Due to the inconsistency in the availability, rabbit anti-human ERα (PLA0113) purchased from Sigma Aldrich (St. Louis, MO, USA) was used in the assay for demonstrating the knockdown efficiency of the transfection of s4824. PLA0113 immunoreacted with the ERα in MCF-7 cell lysate, but not the proteins in MB231 cell lysate [54]. Membranes were blocked in 5% nonfat milk (w/v, in PBS) and incubated with anti-ERα (1:500) and anti-caveolin-1 (1:2000) (in 1% nonfat milk in PBS) at 4^°^C overnight. Immunoreactive proteins were detected and protein band intensities were analyzed as described above.

### ERα activation in a yeast-based ER reporter system (YES)

The activation of ERα was measured by a yeast-based ER reporter gene assay system co-transfected with recombinant human ERα and a LacZ reporter construct [28]. The effects of E2 (0.01-10 nM) and 4-HT (20 μM) were determined to show the responses of known agonist and antagonist, respectively.

### Statistical analysis

The concentration of doxorubicin required to cause a 50% decrease of cell growth (IC_50_) was calculated using GraphFit software (Erithacus Software, Staines, UK). Data analysis by curve fitting generated an estimate and an estimated variance (denoted as ±) of IC_50_ value. The statistical significance of differences between a treated group and the control group was analyzed by the Student’s t test. The differences between > 2 sets of data (control and groups treated with various doses and time periods of oxysterols) were analyzed by one-way ANOVA followed by Dunnett’s test for multiple comparisons. A difference > 5% and p < 0.05 was considered as statistically significant.

## SUPPLEMENTARY MATERIALS FIGURES



## Abbreviations

AKI: Akt inhibitor
BCRP: breast cancer resistant protein
E2: estradiol
ER: estrogen receptor
ERK: extracellular signal-regulated kinase
GAPDH: glyceraldehyde-3-phosphate dehydrogenase
HC: hydroxycholesterol
4-HT: 4-hydroxytamoxifen
7-KC: 7-ketocholesterol
MAPK: mitogen-activated protein kinase
MEK: MAPK/ERK kinase
MRP: multidrug resistance-associated proteins
MTT: 3-(4, 5-dimethyl-thiazol-2yl)-2, 5-diphenyl tetrazolium bromide
PI3K: phosphatidylinositol 3-kinase
P-gp: P-glycoprotein
mTOR: mammalian target of rapamycin
