# Supplementary material for: 7-ketocholesterol and 27-hydroxycholesterol decreased doxorubicin sensitivity in breast cancer cells: estrogenic activity and mTOR pathway
Source: Oncotarget. 2017 Aug 2;8(39):66033–50. doi: 10.18632/oncotarget.19789 (PMC5630390; doi:10.18632/oncotarget.19789)
Supplement: Supplementary file 1 [file oncotarget-08-66033-s001.pdf]

7-ketocholesterol and 27-hydroxycholesterol decreased doxorubicin sensitivity in breast cancer cells: estrogenic activity and mTOR pathway

SUPPLEMENTARY MATERIALS

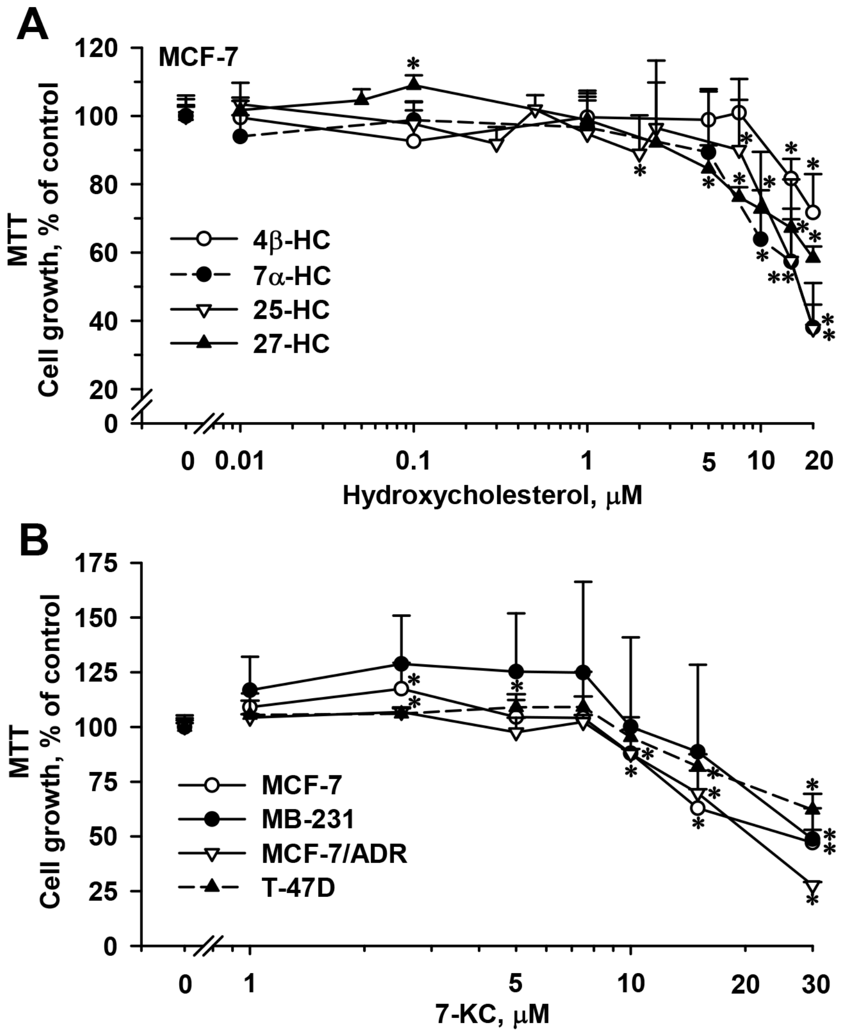

Supplementary Figure 1: Effects of oxysterols on cell growth in breast cancer cell lines. Cell growth was monitored by determining mitochondrial MTT reduction activity. Cells were exposed to oxysterols in 2% FBS-supplemented medium for 48 h. Panel **(A)** shows the effects of hydroxycholesterols (HCs) on the growth of MCF-7 cells and panel **(B)** shows the effects of 7-ketocholesterol (7-KC) on the growth of MCF-7, MB-231, MCF-7/ADR, and T-47D cells. The results are presented as means ± SD of three independent experiments with three determinations within each experiment. *$p < 0.05$.

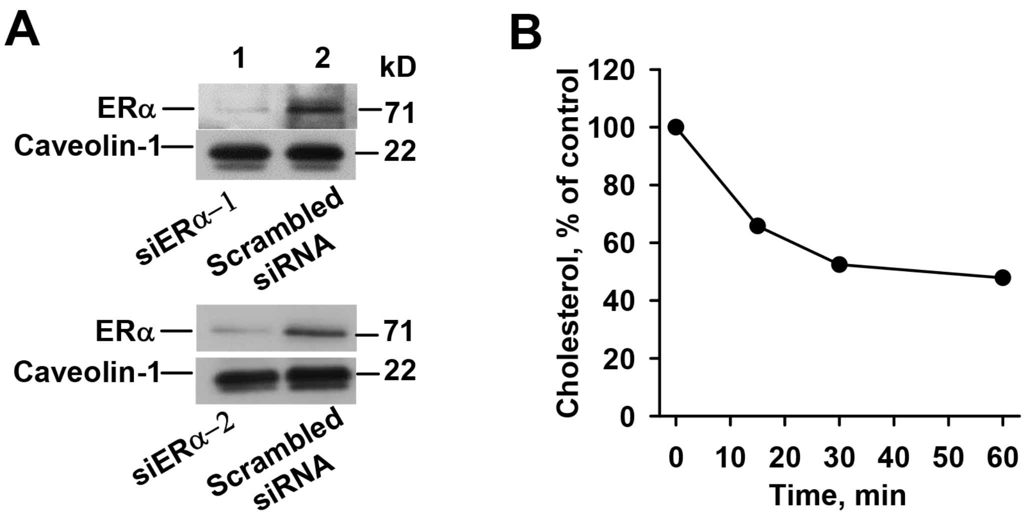

Supplementary Figure 2: The effect of ERα knockdown and methyl-β-cyclodextrin on ERα expression **(A)** and cholesterol level **(B)** in MCF-7 cells, respectively. Panel A shows the representative blot (30 µg crude membrane protein/well) of the immunoblotting analysis of cellular ER protein after transfection with ERα siRNA. Panel B shows the relative cellular cholesterol levels in cells exposed to 10 mM methyl-β-cyclodextrin at 37°C for increasing time-periods.
